# Delicacy and Durability: The Microbiological Sublime

**DOI:** 10.3201/eid2101.AC2101

**Published:** 2015-01

**Authors:** Byron Breedlove

**Affiliations:** Centers for Disease Control and Prevention, Atlanta, Georgia, USA

**Keywords:** art science connection, emerging infectious diseases, Ebola, high-consequence pathogens, Human Microbiome Project, paper sculpture, monochrome, art and medicine, Outbreak, delicacy and durability: the microbiological sublime, Rogan Brown, about the cover

**Figure:**
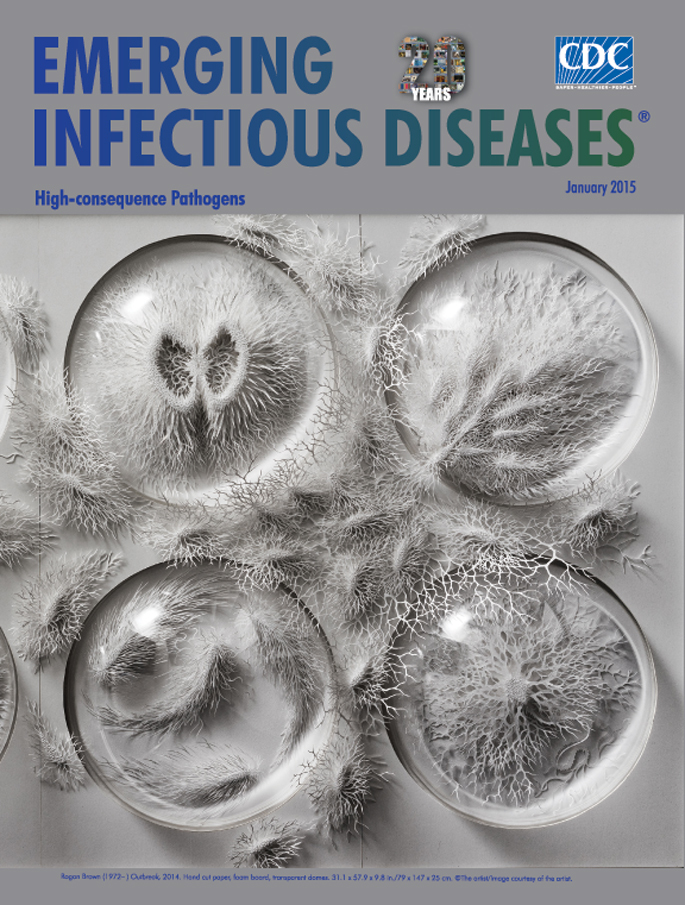
**Rogan Brown (1972–) Outbreak, 2014. Hand cut paper, foam board, transparent domes (31.1 × 57.9 × 9.8 in /79 × 147 × 25 cm)** © The Artist/Image Courtesy of the Artist.

Anglo-Irish artist Rogan Brown, who creates monochromatic sculptures from layers of paper, considers *Outbreak*, this month’s cover image, an exploration “of the microbiological sublime.” Spilling from their petri dishes, overflowing into the space between them, the organisms appear kinetic; the sweep of light and shadow across the image adds depth and dimension to the all-white forms. For full impact, though, this sculpture needs to be viewed in person. In the artist’s words, “Each piece suddenly comes alive when it is placed vertically in the light. Photos only catch them at a certain moment. In reality, the pieces move with the changes in the ambient lighting, so they are always slightly different.” (The quoted text in this essay is from personal communication with Rogan Brown, November 17, 2014.)

To prepare for his work on *Outbreak*, Brown studied myriad photos and diagrams depicting bacteria and viruses. “For the bacteria, I was attracted more by the flagellate forms (salmonella, *E. coli*, *Vibrio cholerae*) simply because they offer a more aesthetically interesting bug-like shape, both beautiful and creepy at the same time, which was the effect I wanted to create. For the viruses, I was looking at influenza and HIV because these are forms that are most accessible and familiar to the public.” He notes that “Although I reference the aesthetics of scientific illustration, diagrams, cut-away models, and so forth, my goal is not to create accurate representations but works of art that create a visual, sensual impact.”

Pathogens and microbes vary immensely in their form, color, and complexity. Stains and dyes are often used to highlight pathogens viewed through microscopes, and those colored, contrasted images may be the first to come to mind. Instead of color, however, Brown focuses on contour and shape, methodically sculpting sheets of paper. For that task, he relies on both traditional tools such as knives and scalpels and modern tools such as laser cutters.

He cites the gluing process as being more stressful that cutting: “Each layer has to be placed with perfect precision on top of the preceding one. There are usually about 8 layers of paper separated by a hidden spacer to create the illusion of floating. The glue does not allow repositioning. I have only one shot, and mistakes are sometimes made.” Brown, who sometimes spends up to 5 months on a project, explains that “The finished artefact is really only the ghostly fossilized vestige of this slow, long process . . . . I have chosen paper as a medium because it captures perfectly that mixture of delicacy and durability that for me characterizes the natural world.”

Originally, Brown planned to create a large installation from which the pathogens would swarm beyond the frame and flow over its walls, spilling onto floor of the gallery space—an idea that may yet see fruition. He found inspiration for this sculpture while attending a microbiology seminar at the Eden Project, a visitor attraction in the United Kingdom; during this seminar, a planned exhibition space focused on the Human Microbiome Project was discussed.

High-consequence pathogens, several of which are highlighted in this issue of *Emerging Infectious Diseases*, provide a rich vein of inspiration for fiction writers and filmmakers. Actual outbreaks caused by such pathogens attract feverish media attention. Brown explains, “I wanted to create this sense of ferocious energy bursting out of the petri-dome, mocking our attempts to control it. Although created before the recent Ebola outbreak, the installation plays on our fears of the microbiological and our sense of powerlessness when confronted by nature.” The frozen tension inherent in *Outbreak* plays to the imagination, starkly capturing the potential genesis of a frightening occurrence.
